# Catalases Induction in High Virulence Pinewood Nematode *Bursaphelenchus xylophilus* under Hydrogen Peroxide-Induced Stress

**DOI:** 10.1371/journal.pone.0123839

**Published:** 2015-04-20

**Authors:** Cláudia S. L. Vicente, Yoriko Ikuyo, Ryoji Shinya, Manuel Mota, Koichi Hasegawa

**Affiliations:** 1 Department of Environmental Biology, College of Bioscience & Biotechnology, Chubu University, Kasugai, Aichi, Japan; 2 ICAAM—Instituto de Ciências Agrárias e Ambientais Mediterrânicas, Departamento de Biologia, Universidade de Évora, Évora, Portugal; 3 HHMI and Division of Biology and Biological Engineering, California Institute of Technology, Pasadena, California, United States of America; Gyeongnam National University of Science and Technology, KOREA, REPUBLIC OF

## Abstract

Considered an EPPO A2 quarantine pest, *Bursaphelenchus xylophilus* is the causal agent of the pine wilt disease and the most devastating plant parasitic nematode attacking coniferous trees in the world. In the early stages of invasion, this nematode has to manage host defence mechanisms, such as strong oxidative stress. Only successful, virulent nematodes are able to tolerate the basal plant defences, and furthermore migrate and proliferate inside of the host tree. In this work, our main objective was to understand to what extent *B*. *xylophilus* catalases are involved in their tolerance to oxidative stress and virulence, using as oxidant agent the reactive oxygen species hydrogen peroxide (H_2_O_2_). After 24 hours of exposure, high virulence isolates of *B*. *xylophilus* could withstand higher H_2_O_2_ concentrations in comparison with low virulence *B*. *xylophilus* and *B*. *mucronatus*, corroborating our observation of *Bxy-ctl-1* and *Bxy-ctl-2* catalase up-regulation under the same experimental conditions. Both catalases are expressed throughout the nematode intestine. In addition, transgenic strains of *Caenorhabditis elegans* overexpressing *B*. *xylophilus* catalases were constructed and evaluated for survival under similar conditions as previously. Our results suggest that catalases of high virulence *B*. *xylophilus* were crucial for nematode survival under prolonged exposure to *in vitro* oxidative stress, highlighting their adaptive response, which could contribute to their success in host conditions.

## Introduction

From 1905 until the present time, pine wilt disease (PWD) has been one of the most threatening diseases for worldwide forests [1]. PWD is considered a complex disease resulting from the interaction of three main elements: the pathogenic agent *Bursaphelenchus xylophilus* (PWN, pine wood nematode), the insect-vector *Monochamus spp*. (responsible for PWN dissemination), and the host tree, mostly from *Pinus spp*. [2–3]. In a *B*. *xylophilus* population, there is intra-specific variability in the ability to cause PWD (virulence) [4]. The existence of different levels of virulence among PWN isolates, and their relation to PWN reproductive ability *in vitro* and *in vivo* were already reported [5–6]. In this context, the low virulence nematodes, often designated as avirulent, show lower reproduction rates and increased generation times that eventually affect their host invasion and colonization [7–9].

In the early stage of PWD, PWN has to cope with different levels of plant immune responses. The first, non-specific, host tree reaction to nematode invasion (or other biotic/abiotic stress) is oxidative burst, an excessive production of reactive oxygen species (ROS). The predominant ROS in plant oxidative burst is H_2_O_2_ [10–11]. H_2_O_2_ is relatively stable, cell wall diffusible, and found to be transversal in different plant-pathogens systems, being a fundamental signal for inducing plant resistance (*i*.*e*. involved in cell-wall reinforcement or induction of defence-related genes in healthy adjacent tissues) [12]. H_2_O_2_ is particularly important since it is extremely reactive and can lead to the formation of radical hydroxyl OH^-^, for which no specific scavenger exists [12]. Generally, parasitic nematodes respond to an oxidant threat by increasing their protective antioxidant enzyme levels [13]. The major antioxidant enzyme families considered are: superoxide dismutases (SOD, EC 1.15.1.1) for conversion of superoxide anion to H_2_O_2_, glutathione peroxidases (GPX, EC 1.11.1.9), catalases (CTL, E.C. 1.11.1.6) and peroxiredoxins (PRDX, EC 1.11.1.15) for the conversion of H_2_O_2_ into water [14]. Furthermore, many parasites employ many enzyme groups under oxidative stress conditions [14]. In plant parasitic nematodes, Molinari and Miacola [15] showed the presence of many antioxidant enzymes and the relation to its lifestyle stages of *Meloidogyne incognita* and *M*. *hapla*, *Globodera rostochiensis and G*. *pallida*, *Heterodera schachtii* and *H*. *carotae*, and *Xiphinema index*. Robertson et al. [16] described an important role of PRDX and GPX in *G*. *rostochiensis*. Bellafiore et al. [17] reported the presence of several detoxifying enzymes, in particular several glutathione S-transferases (GST, EC 2.5.1.18) in the secretome of *M*. *incognita* as means for controlling the global oxidative status and potential nematode virulence. PWN also has an efficient antioxidant system to reduce the deleterious effects of tree oxidative burst [18], mostly present in the nematode secretome [19] and expressed in the cuticle [20].

Pine oxidative burst was found intrinsically related with the production of ROS in the first stages of *B*. *xylophilus* invasion [21–22]. He et al. [23] evaluated the content of H_2_O_2_ of *P*. *thunbergii*, *P*. *massoniana* and *P*. *taeda* at the early stage of *B*. *xylophilus* invasion, and found that H_2_O_2_ content was higher in the susceptible pines than the resistant *P*. *taeda*. In this study, we specifically analyzed the tolerance of *B*. *xylophilus* isolates with different levels of virulence (high and low virulence) under *in vitro* oxidative stress conditions by assessing the effects of H_2_O_2_ exposure to nematode survival, catalase activity, catalase gene expression and their spatial localization. Additionally, we expressed *B*. *xylophilus* in the nematode model *C*. *elegans* to explore the efficiency of *B*. *xylophilus* catalases in promoting survival.

## Material and Methods

### *B*. *xylophilus* isolates and culturing

Two *B*. *xylophilus* isolates with high virulence (Bx Ka4 and Bx T4) and one isolate with low virulence (Bx C14-5) [6] were used in this study. In addition, one isolate of *B*. *mucronatus* (Un1), considered a low virulence nematode, was also used. All isolates were supplied by the FFPRI (Forestry and Forest Products Research Institute). The origin of nematode isolates Bx Ka4 and Bx C14-5 is described by Aikawa and Kikuchi [6]. The isolate Bx T4 was collected from dead *Pinus densiflora* in 1992, in Iwate prefecture, Japan and *B*. *mucronatus* Un1 was obtained from *Monochamus alternatus*, in the Kyoto prefecture.

Nematodes were cultured in *Botrytis cinerea* on autoclaved barley seed at 25°C. Prior to experiments, nematodes were extracted overnight using Baermann funnel technique at 25°C. Nematodes were washed three times with sterilized distilled water with 10 min. centrifugations at 1,000 rpm between washes, surface cleaned with 3% L-lactic acid for 30s, and washed once with sterilized distilled water [9]. Mix-stages nematodes were used in all experiments.

### Oxidative stress tolerance test

H_2_O_2_ was used as oxidative agent at concentrations ranging between 15 and 40 mM for *Bursaphelenchus* isolates. After nematode surface sterilization, the concentration of *Bursaphelenchus* was adjusted to 150 nematodes per 50 μl of sterile water. Mortality of nematodes was scored after 24h-stress exposure. Nematodes were considered dead if no movements were observed after mechanical stimulation. This experiment was repeated three independent times, with two technical replications for each.

### H_2_O_2_ neutralization

H_2_O_2_ neutralization was inferred using an H_2_O_2_ Assay Kit (abcam, Massachusetts, USA). For this purpose, 150 nematodes were exposed for 24h in 15 mM H_2_O_2_. Afterwards, nematodes were pelleted at 1,000 g for 15 min and the supernatant was used for analysis following the manufacture’s protocol. This assay was repeated three independent times with two technical replications.

### Relative gene expression of catalases under oxidative stress

Relative gene expressions of CTL (catalase) enzymes (*Bxy-ctl-1* and *Bxy-ctl-2*), previously predicted by Vicente et al. [24], were analyzed by qRT-PCR using SYBR green assay. Total RNA was extracted from 24h-stressed nematodes (approximately 2,500) in 15 mM H_2_O_2_ using CellAmp Direct RNA Prep Kit for RT-PCR (Takara Bio Inc., Japan) and following manufacture’s instructions. The concentration was quantified using NanoVue plus spectrophotometer (GE Healthcare Life Sciences, USA). Total RNA (adjusted to a concentration of 50 ng/μl) was reverse transcribed using oligo dT primer and PrimeScript RT enzyme from PrimeScript RT reagent Kit (Perfect Real Time, Takara Bio Inc., Japan). qRT-PCR was performed using CFX96 Real-Time (Bio-Rad Laboratories, Inc., California), and SYBR Premix Ex Taq II (Tli RnaseH Plus) kit (Takara Bio Inc., Japan). Actin (*Bxy-act-1*) was used as a reference gene for calculating relative expression levels of CTLs genes [25]. Primers were designed using Prime 3 software [26] and tested for specificity prior to qRT-PCR (Table 1). For each treatment, two experimental replications were conducted with two technical replications each. Controls with no template added were prepared for each qPCR run. Thermal cycling conditions were: initial denaturation at 95°C for 30 sec; 39 cycles of denaturation at 95°C for 5 sec, annealing and extension at 60°C for 30 sec; followed by the melting curve. A single peak at the melting temperature of the PCR-product confirmed primer specificity.

**Table 1:** Characterization of *Bursaphelenchus xylophilus* CTLs, and primers used for CTLs expression analysis.

Gene and Protein DB	ORF^1^	MW (Da)	SignalP^2^	Secretome^3^ (Sec/whole)
***Bxy-ctl-1* (**BUX.s00579.159)	513	58836.42	NO	NO (ND)
***Bxy-ctl-2*** (BUX.s01109.377)	272	54869.71	NO	YES (0.26)
**Primers for qRT-PCR (5’– 3’)**				
***Bxy-ctl-1***	For: GCCAGCGTCTTCAGCAAAGT; Rev: CCAAATTCCGTCATCGGTGT			
***Bxy-ctl-2***	For: CCGACTTCTTTCAACGGAAC; Rev: CCTTCATCGAGCACCTTTTC			
***Bxy-act-1***	For: CATCCTCCGTCTCGACTTGG; Rev: ATGTCACGCACGATTTCACG			
**Primers for *In-situ* hybridization (5’– 3’)**				
**Bxy-CTL-1**	For: CTTCCGATCAACTGCCCCTT; Rev: ATTTAGACAAGGGGCCAGCC			
**Bxy-CTL-2**	For: AGAGACCCCATCCTCTTCCC; Rev: AGCTTCTCCCTTTGCGTTGA			

Sec/whole—Normalized secreted protein/whole proteins; ND—not detected.

^1^ ORF, open reading frame.

^2^Petersen et al. [27].

^3^ Shinya et al. [19].

### *In-situ* hybridization

Single-stranded DNA probes (sense and antisense) labelled with digoxigenin (Roche, United States of America) were used to detect mRNA in mix-stage *B*. *xylophilus* as described by De Boer et al. [28]. Table 1 presents the primers used to generate Bxy-CTL-1 and Bxy-CTL-2 probes (Table 1). As positive control for successful hybridization, ENG (cellulase) probe was also prepared as described by Kikuchi et al. [29]. Images were acquired on an Olympus Bx50 light microscope at 10x-40x magnifications.

### Transgenic *C*. *elegans* construction

*C*. *elegans* culturing and handling were carried out at 20°C as described by Brenner [30]. Strains used in this experiments were N2 (Bristol strain) and DP38 *unc-119(ed3)III*. To make reporter constructs, all PCRs were performed with Takara PrimeSTAR GXL DNA polymerase (Takara Bio Inc., Japan). *B*. *xylophilus ctl-1* and *-2* cDNAs were fused with *C*. *elegans ctl-1* or *-3* promoters and ligated into the *gfp* vector pPD95.77 (kindly provided by A. Fire, Stanford University) with In-Fusion HD Cloning system (Takara Bio Inc., Japan). We cloned a 1,940 bp sequence upstream from the *Ce-ctl-3* start codon for *Ce-Pctl-3*, and a 1,600 bp sequence between *Ce-ctl-3* and *Ce-ctl-1* CDS (coding sequences) for *Ce-Pctl-1*. Fusion genes created in this experiments were *Pctl-1*::*Bxy-ctl-1*::*gfp* and *Pctl-3*::*Bxy-ctl-2*::*gfp*, and PCR primer sequences are listed in Table 2. Each reporter construct (100 μg/ml) was co-injected with an equal concentration of pDP#MM016B into the gonadal arms of *unc-119(ed3)* adult hermaphrodites to obtain KHA149 *{unc-119(ed3)III; chuEx149[Pctl-1*::*Bxy-ctl-1*::*gfp*, *pDP#MM016B]}*, KHA150 *{unc-119(ed3)III; chuEx150[Pctl-1*::*Bxy-ctl-1*::*gfp*, *pDP#MM016B]}*, KHA151 *{unc-119(ed3)III; chuEx151[Pctl-3*::*Bxy-ctl-2*::*gfp*, *pDP#MM016B]}*, and KHA152 *{unc-119(ed3)III; chuEx152[Pctl-3*::*Bxy-ctl-2*::*gfp*, *pDP#MM016B]}*. Successful transgenic *C*. *elegans* were further confirmed by visualization of fluorescence expression patterns with a Nikon SMZ800 dissection microscope equipped with a fluorescence filter and a ZEISS LSM710 confocal laser-scanning microscope.

**Table 2:** List of primers used in *C*. *elegans* constructs.

Genes	Primer 5’– 3’
*Cectl-1Prom_Ifs*	For: CGACTCTAGAGGATCATTGTTTGATATTCAAACTTTTGTA;
	Rev: CGTCATTTTGGTTCTGAAATTTTAGTTAGG
*Cectl-3Prom_Ifs*	For: CGACTCTAGAGGATCCTTCGTCACACTTCTATGGAATCC;
	Rev: GCCATTTTGAAGATTTACTGTTGAATTTCCG
*Bxy-ctl-1_Ifs*	For: CAAACCAAAATGACGGACTTTGCGGCCAATC;
	Rev: CCAATCCCGGGGATCTTGACATCCTCTTGCAATCTCCAC
*Bxy-ctl-2_Ifs*	For: AATCTTCAAAATGGCAAACAACAAGAAGACTGC;
	Rev: CCAATCCCGGGGATCTCAAATGGGCCTTGACCTTCTTGG

### Oxidative stress tolerance in *C*. *elegans*

Synchronized L1 stage *C*. *elegans* were obtained by treating egg-containing/or gravid adults with sodium hypochlorite [30] and allowed to grow on NGM plates seeded with *E*. *coli* OP50 at 20°C for 48 hours until the late L4 stage. L4 stage *C*. *elegans* were washed with M9 buffer and adjusted to 20 nematodes per 50 μl of M9 buffer. Nematodes were transferred to a 96-well plate containing each concentration of H_2_O_2_ (0 to 250 μM). The plate was incubated at 20°C for 24 hours, and then examined for animal viability under a stereomicroscope. Since our transgenic nematodes used in this experiment retained transgenes as an extrachromosomal DNA array, non-transgenic uncoordinated nematodes appeared at a certain proportion (around 30%). We excluded non-transgenic uncoordinated animals when performing this experiment. All tests were carried out at 20°C. This experiment was repeated three independent times, and in each experimental repetition, two technical replications were used.

### Data analysis

Statistical analysis was performed using SPSS version 11.5. One-way ANOVA analysis was performed in order to test if the different *B*. *xylophilus* and *B*. *mucronatus* isolates were equally tolerant to H_2_O_2_ treatments for the parameters: nematode survival percentage, H_2_O_2_ neutralization, and *C*. *elegans* survival percentage. *Post hoc* multi-comparison Duncan test (significance level of 0.05) was used to group significantly different *Bursaphelenchus* sp. isolates in the following parameters: nematode survival percentage, H_2_O_2_ neutralization, and *C*. *elegans* survival percentage. Levene’s Test of Homogeneity was performed a priori to infer ANOVA assumption of homogeneity of variance.

Relative gene expressions of CTLs were analysed using the 2^-ΔΔCT^ method [25]. The data were analysed with C_T_ (cycle threshold) values in normal and stress conditions and using Eq. (1), where ΔΔC_T_ = (C_T,Target_—C_T,Actin_)_normal_-(C_T,Target_—C_T,Actin_)_stress_. The fold change of CTL genes, normalized to *β-act* and relative to the expression at normal conditions, were calculated for each sample using Eq. (1). Data represent the mean ± standard deviation (SD). Statistical differences at 95% and 99% confidence levels were inferred by Student t-test by comparison between relative expression of both CTLs for each *B*. *xylophilus* isolate and the control treatment without H_2_O_2_.

## Results

### Oxidative stress tolerance and H_2_O_2_ neutralisation

**Fig 1:**
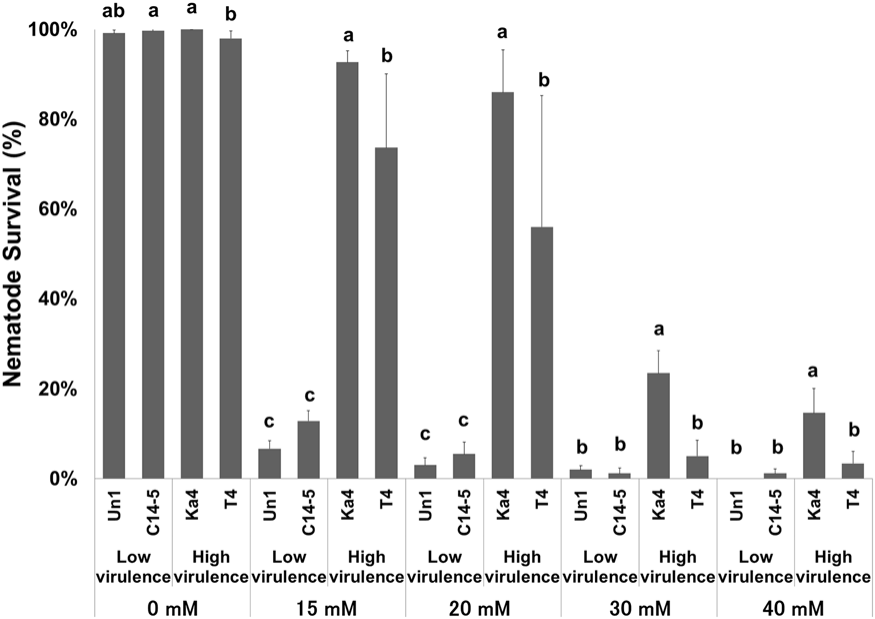
Survival percentage of *Bursaphelenchus* sp. isolates (high virulence *B*. *xylophilus* Ka4 and *B*. *xylophilus* T4, and low virulence *B*. *xylophilus* C14-5 and *B*. *mucronatus* Un1) after 24 hours exposition to H_2_O_2_ conditions (H_2_O_2_ concentrations ranging from 0 to 40 mM). Error bars represent standard deviation. Different letters above the columns indicate significant differences (*p* < 0.05) between *Bursaphelenchus* sp. isolates survival percentages in each H_2_O_2_ treatment, according to *post-hoc* Duncan’s test.

*B*. *xylophilus* (high virulence: Bx Ka4 and Bx T4; low virulence: Bx C14-5) and *B*. *mucronatus* (low virulence: Bm Un1) isolates were tested in increasing concentrations of H_2_O_2_ (0, 15, 20, 30 and 40 mM). After a 24h-exposure to this oxidant, nematode survival was examined (Fig 1). A clear difference between high and low virulence isolates was observed, even in the lowest H_2_O_2_ concentration (15 mM). The isolate Bx Ka4 seemed to be the most tolerant to H_2_O_2_ at all concentrations followed by Bx T4. Statistical differences (*p < 0*.*05*) between Bx Ka4 and Bx T4 were found in all H_2_O_2_ concentrations. Low virulence isolates (Bm Un1 and Bx C14-5) were sensitive to H_2_O_2_, having low survival even in 15 mM H_2_O_2_. No statistical differences (*p > 0*.*05*) were found between isolates.

**Fig 2:**
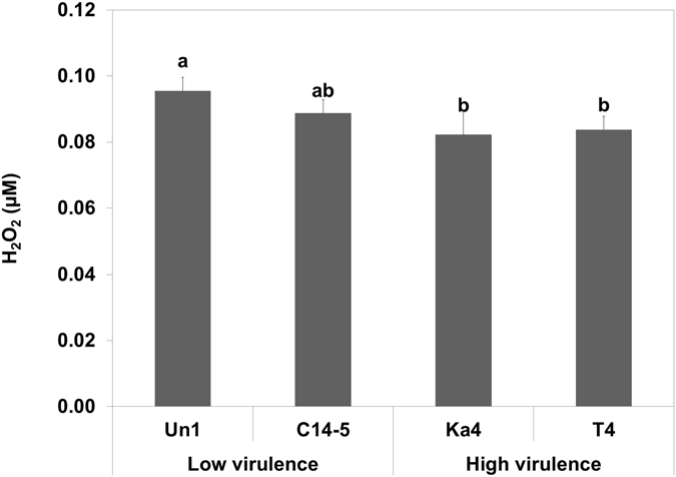
H_2_O_2_ neutralization of of *Bursaphelenchus* sp. isolates (high virulence *B*. *xylophilus* Ka4 and *B*. *xylophilus* T4, and low virulence *B*. *xylophilus* C14-5 and *B*. *mucronatus* Un1) after 24 hours exposition to H_2_O_2_. Error bars represent standard deviation. Different letters above the columns indicate significant differences (*p* < 0.05) between *Bursaphelenchus* sp. isolates H_2_O_2_ neutralization, according to *post-hoc* Duncan’s test.

In terms of H_2_O_2_ neutralization (Fig 2), high virulence Bx Ka4 and Bx T4 were more efficient at H_2_O_2_ reduction (*p < 0*.*05*) in comparison with the low virulence Bm Un1 and Bx C14-5 (no statistical differences between each pair).

### Gene expression analysis

**Fig 3:**
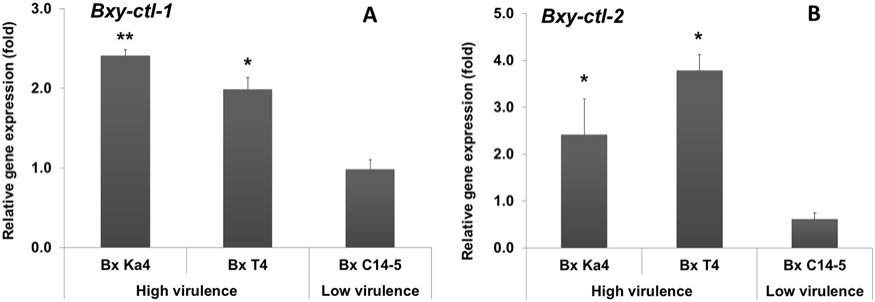
Relative gene expression of *Bxy-ctl-1* (A) and *Bxy-ctl-2* (B) of *B*. *xylophilus* (high virulence isolates Ka4 and T4; low virulence isolate C14-5) after 24 hours exposition to H_2_O_2_. * and ** indicate, respectively, statistical differences at 95% and 99% confidence levels compared to a normalized value of 1.00 for control treatment without H_2_O_2_. Error bars represent standard deviation.

*B*. *xylophilus* catalases (*Bxy-ctl-1* and *Bxy-ctl-2*; Table 1) were previously analyzed and found to be conserved among Nematoda [24]. The CDS of both CTLs were similar between high and low virulence *B*. *xylophilus* isolates (data not shown). Because the lack of *B*. *mucronatus* genome in databases prevented the analyses of gene expression for the Bm Un1 isolate, our studies about levels of virulence will only focus on the Bx Ka4, Bx T4 and Bx C14-5 isolates. Relative gene expression of the CTLs was analyzed after a 24h-exposure to 15 mM H_2_O_2_ (Fig 3). High virulence isolates Bx Ka4 and Bx T4 presented a higher induction of CTL expression than low virulence Bx C14-5. In fact, the relative expression of both *Bxy-ctl-1* and *Bxy-ctl-2* of Bx C14-5 in stress conditions was not significantly different (*p > 0*.*05*) from expression in non-stress conditions. In oxidative conditions, *Bxy-ctl-1* and *Bxy-ctl-2* from both high virulence isolates Bx Ka4 and Bx T4 were significantly induced (*0*.*01< p < 0*.*05*) in comparison with non-stress conditions (Fig 3A and 3B). Both *Bxy-ctl-1* and *Bxy-ctl-2* of Bx Ka4 were 2.5-fold up-regulated. *Bxy-ctl-1* of Bx T4 was significantly induced, nearly 2-fold (Fig 3A). *Bxy-ctl-2* was also significantly up regulated (*p < 0*.*05*) in stress, nearly 4-fold more than in normal conditions (Fig 3B).

**Fig 4:**
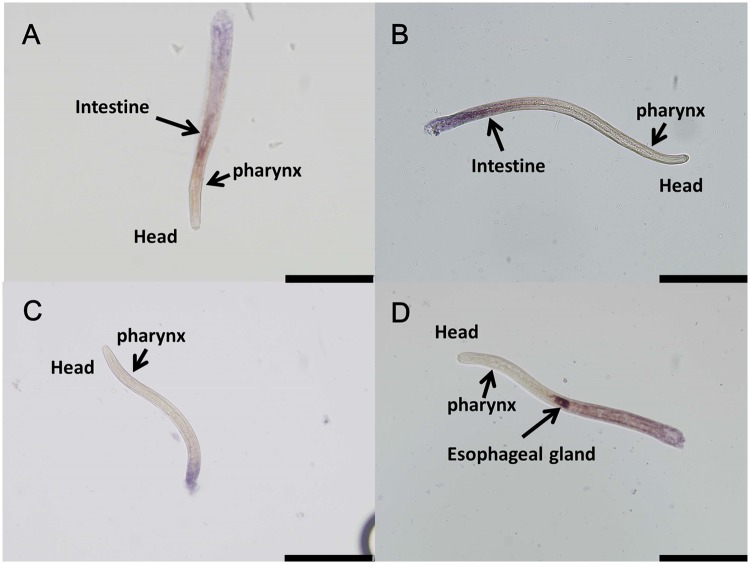
mRNA expression patterns of *Bxy-ctl-1* (A), *Bxy-ctl-2* (B), and *Bxy-eng-1* (D) in *B*. *xylophilus* Ka4. No expression signal was observed with *Bxy-ctl-1* sense probe (C). Light microscope images of *B*. *xylophilus* head region. Scale bars, 100 μm.

*Bxy-ctl-1* and *Bxy-ctl-2* messenger RNAs presented the same expression pattern in the nematode’s intestine (Fig 4A and 4B). *Bxy-eng-1*, profusely expressed in *B*. *xylophilus* oesophageal glands, was used as a positive control (Fig 4D) [29]. No hybridization was observed for the control sense probe of *Bxy-ctl-1* (Fig 4C), *Bxy-ctl-2* or *Bxy-egn-1* (data not shown).

### Transgenic *C*. *elegans* and OS tolerance

Three catalase genes exist in tandem in the *C*. *elegans* genome (*Ce-ctl-3*, *-1*, and *-2*), while two genes exist separately in the *B*. *xylophilus* genome (WormBase, http://www.wormbase.org/). Because *Bxy-ctl-1* and *Bxy-ctl-2* sequences were highly homologous to *Ce-ctl-1* and *Ce-ctl-3* respectively, and *Ce-Pctl-1* is identical to *Ce-Pctl-2*, we constructed transgenic *C*. *elegans* overexpressing *Bx-ctl-1* or *Bxy-ctl-2* with *C*. *elegans* promoters *Ce-Pctl-1* or *Ce-Pctl-3*. We created two independent transgenic lines for each construct; KHA149 and 150 for *Ce-Pctl-1*::*Bx-ctl-1*::*gfp*, and KHA151 and 152 for *Ce-Pctl-3*::*Bx-ctl-2*::*gfp*.

**Fig 5:**
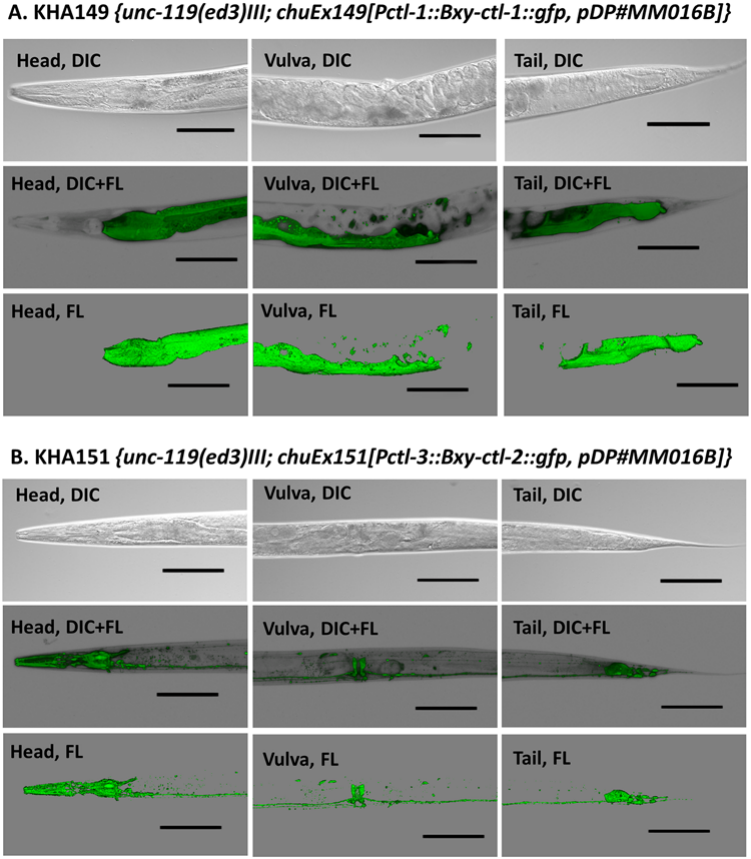
Expression patterns of *Bxy-ctl-1*::*gfp* (A) and *Bxy-ctl-2*::*gfp* (B) in, respectively, transgenic *C*. *elegans* KHA149 and KHA151. Differential interference contrast (DIC) microscope images and, DIC and fluorescence-merged images (DIC+FL) of *C*. *elegans* head, vulva and tail region. Scale bars, 100 μm.

Under the control of *C*. *elegans* promoters, *B*. *xylophilus Bxy-ctl-1* and *Bxt-ctl-2* showed different spatial expression patterns; *ctl-1* was mainly detected in the cytosol of the intestine, and *ctl-2* was detected as high levels along the nervous system and pharynx (Fig 5A and 5B). These expression patterns were the same with those previously reported [31]. Although these transgenic *C*. *elegans* were extrachromosomal lines with unstable transgene transmission, the expression patterns were similar between the transgenic lines KHA149 and 150, and between KHA151 and 152 (data not shown). We used KHA149 and 151 for further experiments.

**Fig 6:**
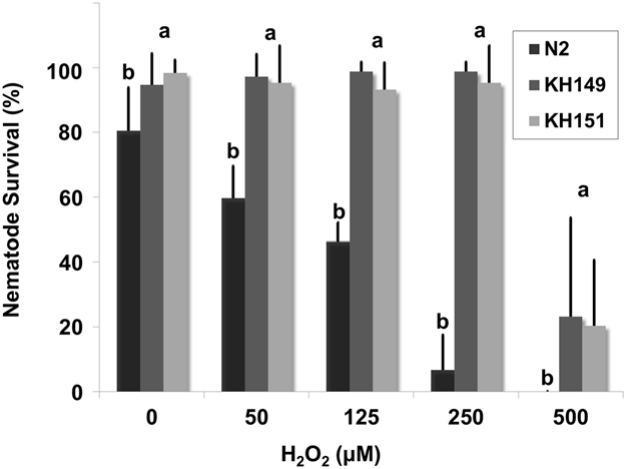
Survival percentage of wild-type (N2) and transgenic (KHA149 and KHA151) *C*. *elegans* after 24 hours exposition to H_2_O_2_ (H_2_O_2_ concentrations ranging between 0 and 500 μM). Error bars represent standard deviation. Different letters above the columns indicate significant differences (*p* < 0.05) between *C*. *elegans N2*, *KHA149 and KHA151* survival percentages in each H_2_O_2_ treatment, according to *post-hoc* Duncan’s test.

To gauge *C*. *elegans* (wild-type and transgenic) tolerance to oxidative stress conditions, they were exposed to H_2_O_2_ concentrations between 50 and 500 μM, substantially lower than the concentrations used for *B*. *xylophilus*. The survival of wild-type *C*. *elegans* N2 decreased significantly (*p < 0*.*05*) with increasing concentrations of H_2_O_2_ (Fig 6). Interestingly, both of the transgenic *C*. *elegans* overexpressing *B*. *xylophilus* catalases were resistant to H_2_O_2_ stress conditions. KHA149 and KHA151, expressing *Bxy-ctl-1* and *Bxy-ctl-2* respectively, were not statistically different (*p > 0*.*05*) in the H_2_O_2_ conditions tested.

## Discussion

Depending on their lifestyle, sedentary or migratory, plant parasitic nematodes possess different repertoires of enzymatic and non-enzymatic molecules involved in oxidative stress metabolism that are a reflection of their pathogenicity [15] In some organisms, like bacteria, these enzymes are defined as virulence factors [32]. Recent work [18] indicates that *B*. *xylophilus* has an enzymatic inventory to counterattack the pine oxidative burst. A total of 12 anti-oxidant enzymes were found in the *B*. *xylophilus* secretome, including PRX, CTL, GST, SOD, nucleoredoxin-like protein and thioredoxin [19]. In the present study, we investigated the tolerance of *B*. *xylophilus* isolates with different levels of virulence (high and low virulence) under *in vitro* oxidative stress conditions using H_2_O_2_ as an oxidizing agent. We were able to observe a contrasting performance between high and low virulence *B*. *xylophilus* and its relative *B*. *mucronatus*, corroborating previous results in other plant parasitic nematodes [33–34]. Emphasizing the results of the oxidative stress tolerance and H_2_O_2_ neutralization tests, the CTL (*Bxy-ctl-1* and *Bxy-ctl-2*) gene expression indicated that high and low virulence *B*. *xylophilus* respond differently towards H_2_O_2_ stress. High virulence nematodes showed an up-regulation of CTL genes under induced oxidative stress conditions, corroborating our preliminary results [24]. In addition, since all *B*. *xylophilus* isolates share protein sequence similarity between CTLs, the contrasting responses to H_2_O_2_ stress may indicate different regulation in the detoxification of ROS. This correlation between catalase levels and *in vitro* H_2_O_2_ tolerance has also been described in other parasitic nematodes [35–36]. Previously, Shinya et al. [20] showed the accumulation of anti-oxidant and detoxifying enzymes on the body surface of the nematode. Here, we were able to determine that both *B*. *xylophilus* catalases (*Bxy-ctl-1* and *Bxy-ctl-2*) are expressed in the intestine.

We also accounted for other antioxidant enzymes (SODs and GXPs) under H_2_O_2_ stress (S1 Fig), and found that, in our experimental conditions, only catalase expressions were significant. Although we could ascertain the importance of CTLs (*Bxy-ctl-1* and *Bxy-ctl-2*) in the protection against H_2_O_2_ induced-stress, we cannot rule out the effect of other antioxidant proteins in their response. Another important observation in this study is the maintenance of CTL induction in high virulence *B*. *xylophilus* even after 24h of H_2_O_2_ exposure. Kotze [37] observed similar CTL induction in parasitic nematode *Haemonchus contortus* against *in vitro* H_2_O_2_ stress. This feature is crucial and highlights the adaptive response and antioxidant protection of high virulence *B*. *xylophilus* in host conditions. Further studies are still needed to completely understand the role of all protectant molecules (enzymatic or non-enzymatic) of *B*. *xylophilus* against the massive ROS production of susceptible pines.

The regulatory pathways of xenobiotic degradation of *C*. *elegans* are conserved in *B*. *xylophilus* [18]. Hence, We constructed transgenic *C*. *elegans* overexpressing both *Bxy-ctl-1* and *Bxy-ctl-2*. Since we previously have shown that *Bxy-ctl-1* sequence was more similar to *Ce-ctl-1* and *Ce-ctl-2*, and *Bxy-ctl-2* to *Ce-ctl-3* [24], we chose *C*. *elegans Pctl-1 to regulate Bxy-ctl-1* and *C*. *elegans Pctl-3* to regulate *Bxt-ctl-2*. Our results showed that subcellular localization of both catalases is in accordance with *C*. *elegans ctl-1* and *ctl-3* promoter expression as shown by Petriv and Rachubinski [38]. Furthermore, Oláhova et al. [31] have also shown a decrease in survival rate of wild-type N2 to 1 mM H_2_O_2_ only 10 hours after treatment, which support our selection to test 50–500 μM H_2_O_2_ as experimental concentrations. *C*. *elegans* contains three catalases, *Ce-ctl-1* (cytosolic), *Ce-ctl-2* (peroxisomal) and *Ce-ctl-3* (neurons, muscles and pharynx), which present different activities, with CTL-2 being responsible for 80% of total catalase activity [31]. Expressing exogenous *B*. *xylophilus* catalases, transgenic KHA149 and KHA151 animals were able to withstand the H_2_O_2_ stress up to 500 μM, supporting the efficiency of *B*. *xylophilus* catalases in H_2_O_2_ neutralization.

## Supporting Information

S1 Fig Relative gene expression of SODs (*Bxy-sod-1*; *Bxy-sod-2*; *Bxy-sod-3*) and GXPs (*Bxy-gxp-1*; *Bxy-gxp-2*; *Bxy-gxp-3*) of *B*. *xylophilus* (high virulence isolates Ka4 and T4; low virulence isolate C14-5) under oxidative stress conditions. Error bars represent standard deviation.
(TIF)
