# Supplementary figures and images for: Catalases Induction in High Virulence Pinewood Nematode Bursaphelenchus xylophilus under Hydrogen Peroxide-Induced Stress
Source: PLoS One. 2015 Apr 20;10(4):e0123839. doi: 10.1371/journal.pone.0123839 (PMC4404050; doi:10.1371/journal.pone.0123839)

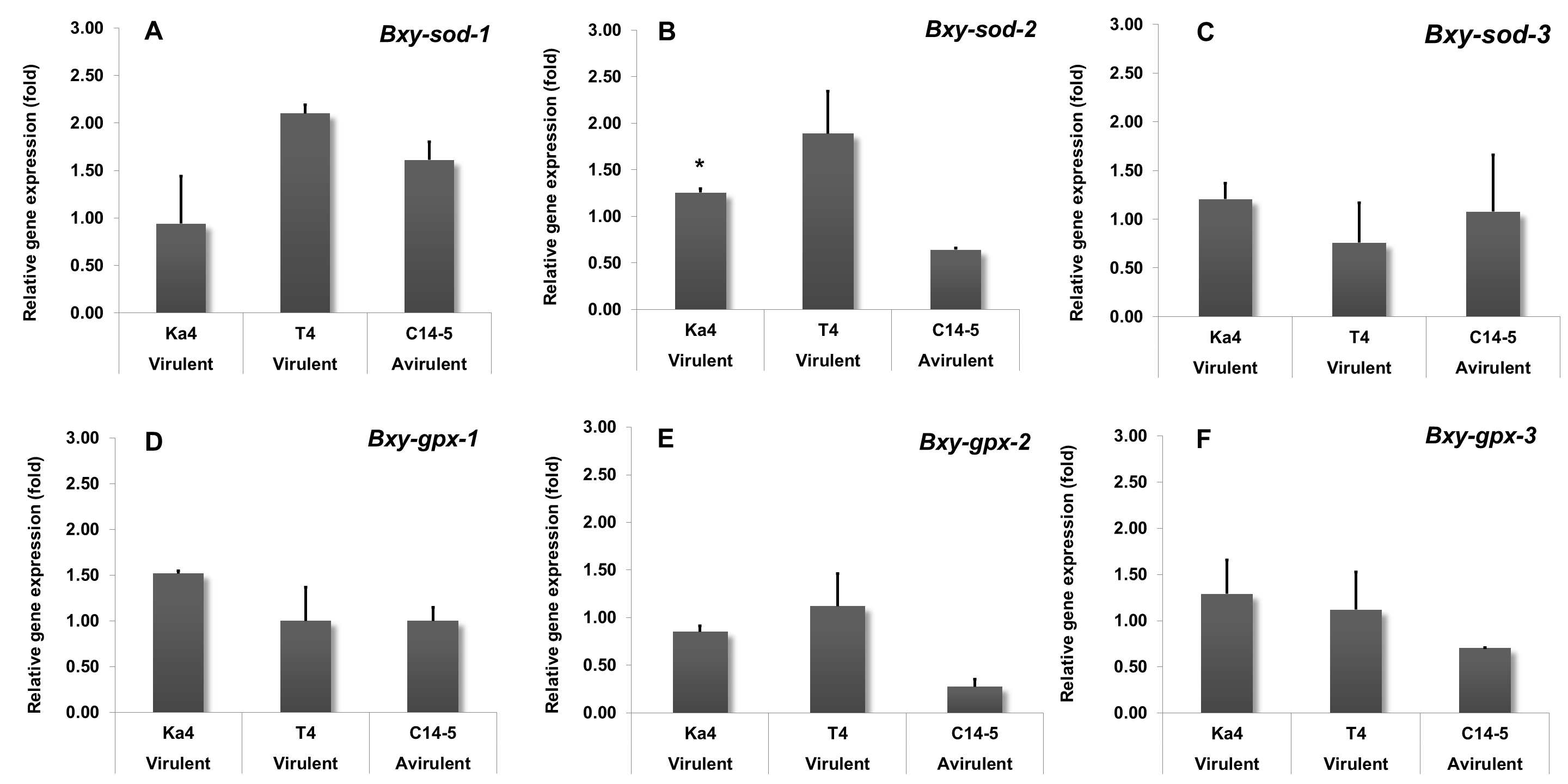

Supplement: S1 Fig — Error bars represent standard deviation. (TIF) [file pone.0123839.s001.tif]
